# A Theoretical Lower Bound for Selection on the Expression Levels of Proteins

**DOI:** 10.1093/gbe/evw126

**Published:** 2016-06-11

**Authors:** Morgan N. Price, Adam P. Arkin

**Affiliations:** Environmental Genomics and Systems Biology, Lawrence Berkeley National Lab

**Keywords:** gene expression levels, metabolic models, microbiology

## Abstract

We use simple models of the costs and benefits of microbial gene expression to show that changing a protein’s expression away from its optimum by 2-fold should reduce fitness by at least 0.2·P, where *P* is the fraction the cell’s protein that the gene accounts for. As microbial genes are usually expressed at above 5 parts per million, and effective population sizes are likely to be above 10^6^, this implies that 2-fold changes to gene expression levels are under strong selection, as $N_{e}\cdot s\gg 1$, where *N_e_* is the effective population size and *s* is the selection coefficient. Thus, most gene duplications should be selected against. On the other hand, we predict that for most genes, small changes in the expression will be effectively neutral.

## Introduction

Every cell contains hundreds or thousands of different proteins, and the abundance of these proteins varies by orders of magnitude (Lu et al. 2007; Ingolia et al. 2009; Li et al. 2014). A recurring question is whether natural selection will drive these abundances to their optimal levels. Because single-nucleotide mutations in promoter regions can achieve a wide range of changes to expression levels (Shultzaberger et al. 2010), optimal expression should evolve rapidly if the selection of expression levels is strong.

For most genes, 2-fold changes in expression can be tolerated. In diploid organisms, loss-of-function mutations are often recessive, which implies that the loss of one of two copies of a gene has little consequence. In the budding yeast *Saccharomyces cerevisiae*, diploid strains that have just one functional copy of a gene are almost always viable in rich or minimal media, and for just 3% of genes does this reduction in copy number lead to a measurable reduction in growth rate (Deutschbauer et al. 2005). Testing a larger number of conditions increases the proportion of haploinsufficient genes to 20% (Delneri et al. 2007). In contrast, the complete loss of almost any yeast gene causes a measurable reduction in growth rate in some laboratory condition (Hillenmeyer et al. 2008).

These screens are only sensitive down to relative changes in the growth rate of 1% or so, but natural selection will remove a deleterious allele from a microbial population even if its selective advantage is on the order of $10^{-6}$. Under the nearly neutral theory of molecular evolution, the critical question is whether $N_{e}\cdot |s|>1$, where *N_e_* is the effective population size and *s* is the difference in fitness or the relative change in the growth rate. (The effective population size describes the importance of genetic drift in an evolving population (Charlesworth, 2009).) For both yeast and bacteria, we expect that *N_e_* is around 10^6^ or 10^7^ (Tsai et al. 2008; Price and Arkin, 2015). Thus, laboratory measurements are not sensitive enough to tell us if there is strong selection on protein levels.

Another way to test whether a trait is under strong selection is to look at the variation of the trait within a population or the divergence of the trait between closely-related species. Recently, ribosomal profiling has been used to compare the rates of translation and transcription in various yeasts of the genus *Saccharomyces*. A comparison of related yeast species found a surplus of opposing changes to transcript abundance and to the efficiency of each transcript’s translation, such that mRNA levels vary more than protein production does (McManus et al. 2014). These results are consistent with stabilizing selection on the expression of many proteins. However, a similar study that compared two different strains of *S. cerevisiae* did not find a surplus of opposing changes (Albert et al. 2014).

A related approach has been to study the fate of duplicated genes. For example, in several organisms, the rate of gene duplications in the laboratory is much higher than was expected from evolutionary studies, which suggests that most duplicates are removed by natural selection (Katju and Bergthorsson, 2013). As another example, a whole-genome duplication creates two identical copies of all genes. The loss of one of the paralogs would cut the gene expression in half, and such a loss is less likely to occur in genes that are highly expressed (Gout et al. 2010; Gout and Lynch, 2015). This suggests that the selective disadvantage of a 2-fold change in expression is stronger for more highly-expressed genes (Gout et al. 2010). Furthermore, once the expression levels of the paralogs have diverged, so that one is expressed more highly than the other, a paralog is more likely to be lost (Gout and Lynch, 2015). This suggests that smaller changes in expression levels (due to loss of the more weakly-expressed paralog) are under weaker selection.

Finally, various theoretical models have been used to predict the impact of changes to expression levels. First, the growth rate of a microbial cell can be thought of as the flux through a complex network of metabolic pathways. Naively, one might expect a metabolic pathway to have a rate-limiting step, such that changes in the expression of that enzyme would have a large impact on the flux through the pathway. However, metabolic pathways usually do not behave this way – instead, control is distributed across all of the reactions (Kacser and Burns, 1981; Fell and Cornish-Bowden, 1997). Given a large number of reactions, this implies that changing the expression of any one gene will have small effects (as seen in studies of haploinsufficiency) – but how small?

Second, Wagner proposed that for most genes in *S. cerevisiae*, a 1% increase in relative expression would be selected against (Wagner, 2005, 2007). However, Wagner’s analysis considered that an increase in expression would pose a cost, but did not consider that a small increase in a protein’s expression would have some benefit (even if less than the cost), due to the increased activity. So we suspect that selection on small changes in expression are much weaker.

Third, Gout and colleagues proposed a cost-benefit model of gene expression. To explain why selection on gene expression levels seems to be stronger for more highly-expressed genes, they assumed that the benefit increases linearly with expression level, while the cost increases more quickly than linearly (Gout et al. 2010). Their model has free parameters, so it does not constrain the absolute strength of selection on gene expression levels. Also, we will show that the linear benefit and the super-linear cost are not realistic for most proteins.

Our approach is to examine the impact of changes in gene expression levels in simple models of metabolism or growth. We identify a lower bound on the cost of changing a protein’s expression away from its optimum. For 2-fold changes in expression, the reduction in fitness is at least 0.2·P, where *P* is the fraction of all protein that the gene accounts for. Although this effect is small, it is likely to be significant for evolution. On the other hand, our models suggest that small changes in expression, such as the 1% relative change that was proposed to be significant in yeast (Wagner, 2005), may be effectively neutral for most genes.

## Results

We will study the cost of changing a protein’s expression away from its optimum in several different models. We will start with the simplest possible model – a linear metabolic pathway without enzyme saturation, so that growth is equivalent to the flux through the pathway. We will show that this model is approximately equivalent to a simple cost-benefit form (Cherry, 2010), and we will focus our discussion on this cost-benefit form. Of course, the actual growth of a cell is far more complicated than this linear pathway, and involves saturating enzymes, protein synthesis, multiple-input and multiple-output reactions, metabolic cycles, and metabolic regulation such as end-product inhibition. We will study models that include saturating enzymes or the assembly of amino acids into proteins. Finally, we will use metabolic control analysis (Kacser and Burns, 1981) to show that other complications in metabolism are not likely to affect our conclusions.

## The Cost-Benefit Form

Consider a linear pathway with reversible enzymes and no enzyme saturation. In the Models section, we show that given such a pathway, the dependence of the steady-state flux on a focal protein’s expression level can be decomposed into the benefit minus the cost. Specifically, assume that the total protein concentration is held fixed and let *P* be the fraction of all protein that is the focal protein. If *P* changes, then we assume that the expression of the other proteins is multiplied by 1−P to compensate. (For a theoretical argument for why the total concentration of macromolecules within a cell is kept constant, see Dill et al. (2011).) For example, if the expression of the focal protein increases from 1% to 2%, then expression of all other proteins would change by a factor of 0.98/0.99 or roughly a 1% reduction. Then the dependence of the flux *F* on *P* is given by

$$F\propto \frac{P}{K+\frac{P}{1-P}}\approx \frac{P}{K+P}-P$$

where *K* is the investment required to obtain the half-maximum benefit, and the approximation is accurate if P≪1, as is true for all natural proteins. This equation shows the benefit minus the cost. Also, since the flux is maximized when $P\approx \sqrt{K}$, and P≪1, we can assume that the saturation constant K≪1. Then, at optimal *P*, benefit - cost ≈1. If we consider the relative flux as equivalent to fitness, then the change to benefit - cost is proportionate to and roughly equal to the selection coefficient.

**Fig. 1.**
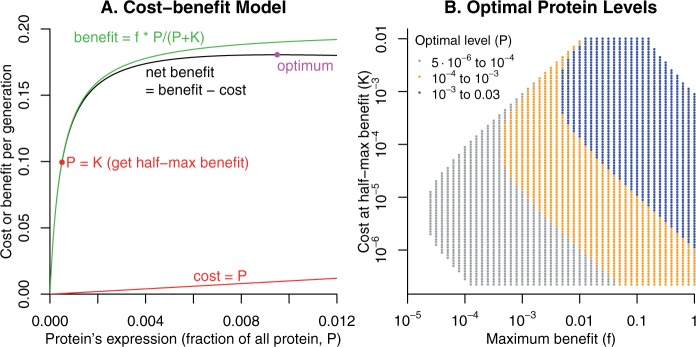
The cost-benefit form. (A) The net benefit as a function of the protein’s expression level. In this example, the maximum benefit is *f* = 0.2 and the cost at half-max expression is $K=5\cdot 10^{-4}$. (B) The optimum expression level as a function of *f* and *K*. Points are only shown if the optimal expression level is between $5\cdot 10^{-6}$ and 0.03. Note log axes.

To account for nonessential proteins, we scale the benefit by a new parameter (*f*) which represents the maximum possible benefit (fig. 1A). To see why the saturating benefit is plausible for a nonessential protein, consider a protein that repairs a rare form of DNA damage that would prevent DNA replication. A low level of expression of the enzyme will allow the cell to continue growing and will be highly beneficial when the damage occurs. Increasing levels of enzyme will give small decreases in the time that the cell waits for the damage to be repaired until it can start growing again – still beneficial, but far less so. The cost-benefit form has been used to explain why the evolutionary rate of a protein sequence depends on the protein expression level (*P*) rather than its importance for fitness (*f*) (Cherry, 2010).

## The Cost of Microbial Gene Expression

The cost-benefit form implies that the cost (the reduction in fitness) of expressing a useless protein is roughly the same as the protein level (as a fraction of all protein). (Note that both terms are dimensionless.) Similarly, in models of microbial growth that includes protein synthesis as well as metabolism, the cost of useless protein is equal to its proportion (Weiße et al. 2015) or to a small multiple (Scott et al. 2010). Furthermore, empirical studies are consistent with the simple theory that the cost of useless protein is roughly the protein level. In studies of fast-growing cells that overexpress proteins that do not benefit the organism, the reduction in relative growth rate is roughly 1-2 times the fraction of all protein that the useless protein accounts for (Shachrai et al. 2010; Scott et al. 2010; Tomala and Korona, 2013; Kafri et al. 2016).

In contrast, Gout and colleagues (Gout et al. 2010) assumed a super-linear cost of expressing additional protein. Metabolic models do imply a super-linear cost for highly-expressed proteins, but this effect is tiny unless P≥0.1, where *P* is the fraction of all protein in the cell that this gene accounts for (see Models). Such high expression occurs in few if any genes. For instance, in rich media, essential proteins in the model bacterium *Escherichia coli* are expressed at $P=6\cdot 10^{-6}$ to 0.03 (combining Li et al. (2014); Kato and Hashimoto (2007)). Similarly, essential proteins in *S. cerevisiae* are expressed at $P=7\cdot 10^{-7}$ to 0.02 (combining Deutschbauer et al. (2005); Ingolia et al. (2009)). So we doubt that the super-linear cost is significant in practice.

## The Benefit of Microbial Gene Expression

The shape of the benefit, as a function of the expression level, has been studied by using synonymous mutations to alter the expression level of the lactose-degrading enzyme LacZ in *E. coli* and by using a nonmetabolizable inducer to prevent the metabolism from influencing expression (Eames and Kortemme, 2012). (To control for the cost of expression, these experiments compared growth in the presence of lactose to growth in the absence of lactose.) These experiments show a saturating (Michaelis-Menten like) benefit, which is consistent with the cost-benefit form.

The maximum benefit (*f*) might range from $f=10^{-5}$, for a protein with very subtle benefits that could still be selected for, to *f* = 1, for essential proteins. To see why very low benefits are possible, consider that a benefit of $10^{-5}$ with a low cost and $N_{e}\approx 10^{6}$ would imply $s\approx 10\cdot N_{e}$. If mutation rates are equal in both directions then $s>N_{e}$ is sufficient to maintain the preferred allele in the population with high probability. But a gene is much more likely to be inactivated by mutation than to revert. Because that the typical protein-coding gene has on the order of 1,000 coding nucleotides, we expect that the ratio of the two mutation rates is around 1,000 fold. Then the selection coefficient necessary to maintain the allele is increased by log(1000)≈7 fold (Bulmer, 1991), i.e., around $10^{-5}$ not $10^{-6}$. Also, in very large populations, the frequency of deleterious alleles would be roughly μ/s, where *μ* is the mutation rate, or about $10^{-7}$ for loss of a gene. This again implies that most members of the population could retain a gene with a net benefit of around $10^{-5}$. Although such subtle benefits are theoretically possible, in both yeast and bacteria, most genes have phenotypes in the laboratory (Hillenmeyer et al. 2008; Deutschbauer et al. 2014), which implies that most proteins have *f* > 0.01 at least in some conditions.

The expression level required for half-max benefit (*K*) must be at least 1 copy per cell, which is around $2\cdot 10^{-7}$ for *E. coli* (Li et al. 2014), so we use this as a lower bound. But it is possible that proteins in larger microorganisms such as yeast could have lower costs if they are specific to a small compartment.

**Fig. 2.**
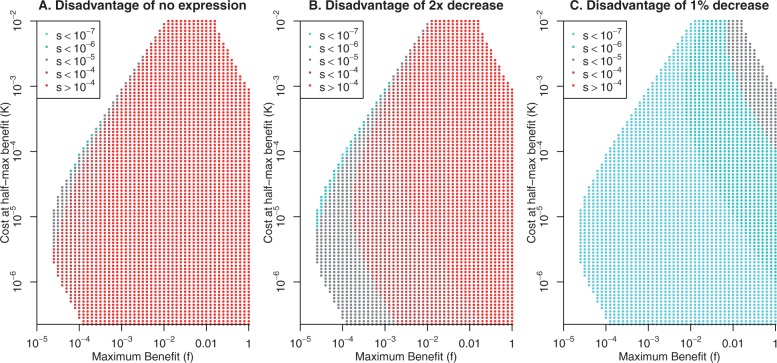
Selection against changes in expression. Using the cost-benefit form, we estimated the selection against a reduction in the protein’s expression from the optimum level to (A) zero expression, (B) half of optimal expression, or (C) 99% of optimal expression. Only parameter settings where the optimum protein expression is between $5\cdot 10^{-6}$ and 0.03 are shown.

The protein can benefit the cell if *f*>* K*, and given this constraint, a wide range of values of *f* and *K* are consistent with optimal expression levels of $5\cdot 10^{-6}<P<0.01$ (fig. 1B). We consider this as the realistic range of expression levels because it accounts for over 95% of proteins that are essential or important for fitness in either *S. cerevisiae* (Deutschbauer et al. 2005; Ingolia et al. 2009) or *E. coli* (Baba et al. 2006; Li et al. 2014; Price et al. 2016). Except when *f* and *K* are of about the same order of magnitude, the net benefit of the protein is close to the maximum benefit *f*. Thus, most parameters show strong selection for the protein, with $s>10^{-4}$ (fig. 2A), which seems realistic.

## The Disadvantage of Changing a Protein’s Expression from Its Optimum

Suppose that a protein is expressed at optimal levels, and then a mutation alters its expression by a small fraction. In the cost-benefit form, the selective disadvantage is roughly proportionate to the square of the fraction times the protein’s level *P_opt_* (see Models). For example, a 2-fold reduction in expression would reduce fitness by roughly $0.5\cdot P_{opt}$, as would a 2-fold increase in expression. A 1% decrease or a 1% increase would reduce fitness by roughly $0.0001\cdot P_{opt}$. These approximations are accurate to within 2-fold as long as the protein’s maximum benefit is an order of magnitude higher than the cost at half-max benefit (f>10·K). Although proteins with high relative costs are theoretically possible, most proteins have modest expression, or *P* < 0.001 (Ingolia et al. 2009; Li et al. 2014), and have significant effects on fitness in laboratory conditions (Hillenmeyer et al. 2008; Deutschbauer et al. 2014), which implies that benefit - cost > 0.01. These observations are not compatible with high relative costs. In any case, exact results for the selective impact of reducing a protein’s expression by 2-fold or by 1% are shown in figure 2b and *c*. Also, as proposed by Gout et al. (2010), the selective disadvantage of a change in expression does not depend primarily on the importance of the gene for fitness. For example, the selection against a 2-fold reduction in expression is much more strongly correlated with the optimal expression level (*r* = 0.98) than with the benefit minus the cost (*r* = 0.48).

Although this model suggests that a 1% change in expression would reduce fitness by just $0.0001\cdot P_{opt}$, this should be viewed as a lower bound. The benefit term in the cost-benefit form saturates very slowly, which is unrealistically slow for most genes. To see this, consider the optimal expression of an essential protein in the model bacterium *E. coli*. Given our cost-benefit form, the protein’s optimal expression level is approximated by $P_{opt}\approx \sqrt{K\cdot f}$. Given $K>2\cdot 10^{-7}$ and *f* = 1, then the optimal expression level would be at least $4.5\cdot 10^{-4}$. But the majority of essential proteins in *E. coli* are expressed well below this prediction, with a median expression of $1.6\cdot 10^{-4}$ (Kato and Hashimoto, 2007; Li et al. 2014). Below, we show that in a growth model, optimal expression levels are much lower, yet the selection on small changes in expression remains weak.

## Selection on the Expression Levels of Heteromeric Complexes

A major limitation of our models is that they implicitly assume that each enzyme is monomeric or homomeric. Our proposed lower bound will not apply proteins that form stable heteromeric complexes. For example, if the two subunits of a heterodimeric enzyme are expressed at optimal and equal levels and then the expression of one subunit increases, then the concentration of active enzyme might not increase at all. (This is true as long as the two subunits bind each other tightly.) Since there is no incremental benefit to the increased expression, the reduction in fitness is the same as the increase in the cost term, or |s|≈|ΔP|. This implies that even small increases in expression might be strongly selected against, as proposed by Wagner for all proteins in yeast (Wagner, 2005, 2007). Conversely, if the expression of one subunit drops, then some of the other subunits will be useless, so again selection on changes to expression will be stronger. Indeed, in *S. cerevisiae*, genes with detectable haploinsufficiency are often found in heteromeric complexes (Papp et al. 2003; Deutschbauer et al. 2005), and over half of the ribosomal proteins are haploinsufficient in rich media (Deutschbauer et al. 2005).

Although heteromeric complexes are a major exception for our models, they account for a small fraction of proteins. In a metabolic model of *S. cerevisiae*, 12% of the enzymatic reactions or transport reactions that are linked to a gene are associated with heteromers (Heavner et al. 2013).

In bacteria and archaea, proteins that physically interact are often found in operons (Dandekar et al. 1998). If the entire complex is encoded by one operon, then many mutations will alter the expression of all the components of the complex in unison (i.e., a mutation to the operon’s promoter, or the duplication of the entire operon). In these cases, there is an incremental benefit to the excess expression, our models apply, and we predict that selection would be relatively weak. In contrast, mutations to a ribosome binding site for one of the genes would affect the expression of just one component and would be under strong selection as envisioned by Wagner.

We also speculate that the expression levels of heteromeric complexes might not evolve to their optima if multiple mutations in different promoters are required to see a benefit. In bacteria and archaea, this could be another reason why operons that are conserved over long spans of evolutionary time tend to encode proteins that physically interact (Dandekar et al. 1998).

## Disadvantage of Nonoptimal Expression in Other Metabolic Models

**Fig. 3.**
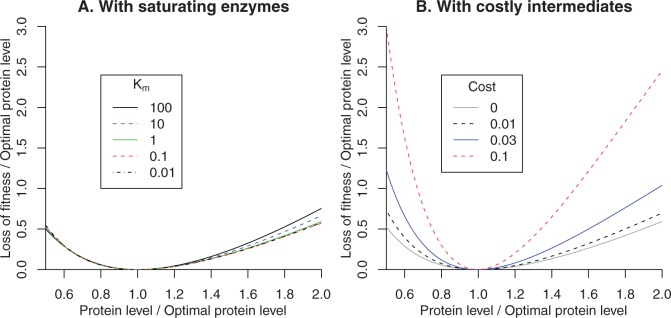
The impact of changes in expression levels when enzymes saturate or metabolic intermediates are expensive. For a two-step enzymatic pathway S↔I↔E, we show how the flux varies with the fraction of protein that is allocated to the first step. The *x* axis shows the first protein’s level relative to the optimum level for that protein ($x=P/P_{opt}$). The *y* axis shows the relative reduction in flux divided by the optimum protein fraction ($y=s/P_{opt}$). In (A) we consider a range of values for the enzyme’s Michaelis constant (*K_m_*). In (B), we consider the cost of intermediates, with *K_m_* = 1 mM. The intermediates reduce flux by diluting out both enzymes, or in other words the flux is divided by 1+I·cost.

We considered several refinements to our model to make it more realistic and to see if the effect of small changes in expression was increased. First, the saturating term in the cost-benefit form was derived by assuming that the enzymes are not saturated by their substrates, which is not realistic. So we considered a simple two-step pathway with reversible Michaelis-Menten kinetics, of the form S ⟷ I ⟷ E, where S is the substrate, I is an intermediate, and E is the end product. We assumed that the substrate concentration is 2 mM, the end product is 1 mM, and that both reactions are mildly favorable with equilibrium constants of 10. We focused on the expression of the first protein in the pathway, and so that its expression is reasonably moderate, we assumed that the first enzyme is 100 times more active (per unit mass) than the second enzyme. We set all of the enzyme’s Michaelis constants to be the same (*K_m_*), and we varied *K_m_* from 0.01 to 100 mM. We found that the cost of a 2-fold change in expression was roughly 0.5·P regardless of the choice of *K_m_* (fig. 3a). We found similar results if we focused on the second step of the pathway instead (not shown).

A second omission in our model is the cost of high concentrations of intermediates. Once the benefit term is nearly saturated, increases in enzyme levels still yield increasing benefits because the build-up of intermediates increases flux through downstream reactions. (The increased level of enzyme may also lead to a lower concentration of its substrate and hence an increase in the net flux of upstream enzymes.) However, high concentrations of intermediates might be costly because of dilution, because of the cost of producing this additional biomass, or because they are toxic. Even if they are not toxic, we expect that intermediates dilute out other components because otherwise the concentration of water would drop, which might cause enzymes to misfold or might reduce diffusion rates (this is similar to the argument of (Dill et al. 2011)). In the above simulations with *K_m_* = 1, the optimal expression level of the first enzyme is 9.4%, and the concentration of intermediates is 9.8 mM. Let us suppose that these intermediates dilute the enzymes, with the molecular weight of the intermediate being 100 times less than that of an enzyme. We also assume that the total concentration of protein monomers is 4 mM (BioNumbers 104726, Bremer and Dennis (1996); Milo et al. (2010)). Then the optimal expression is reduced to 8.8%; the optimal concentration of the intermediate drops to 9.5 mM; and the relative impact of a 2-fold increase (or decrease) in expression away from the optimum increases slightly, from $s/P_{opt}=0.59$ (or 0.53) to $s/P_{opt}=0.61$ (or 0.57). Even a dramatic increase in cost, corresponding to an intermediate that weighs 1/10th as much as the enzymes, only increases the selective disadvantage of a 2-fold change by about two fold (fig. 3b). Similarly, with the high cost, the impact on the fitness of a 1% change in expression is about 2-fold higher than in a model with no cost of intermediates (roughly 0.0002·P instead of 0.0001·P). Varying *K_m_* from 0.001 to 10 made little difference to this result (s/P=0.00014 to 0.00027).

Our interpretation is that the cost of intermediates can strengthen the selection on small changes in protein levels, but only if the intermediates are extremely expensive. Such a high cost for metabolic intermediates is not realistic given the cost of biomass or dilution, because metabolites weigh too little relative to enzymes. But if the intermediate inhibits other enzymes or are otherwise toxic, then more stringent selection of protein levels may occur.

Finally, consider the possibility of redundant enzymes. For example, suppose that after a gene duplication, the two paralogs have similar expression levels and identical molecular functions. In this case, altering the expression of one paralog by 2-fold will alter the total enzyme concentration by 25%, and will affect the growth rate by roughly $0.25^{2}\cdot P_{tot}=0.05\cdot P_{tot}=0.1\cdot P$. A similar argument shows that selection on the expression of parallel pathways could be relaxed. Although this is an exception to our proposed lower bound, we expect that most putatively redundant genes are not maintained unless the individual genes have significant advantages under some conditions.

## Disadvantage of Nonoptimal Expression in a Growth Model

So far we have discussed linear metabolic pathways and the cost-benefit form that was derived by considering a linear pathway. We next considered a simple model of the assembly of amino acids into proteins. This model captures two key aspects of cells that are missing from a linear metabolic pathway: proteins that make more proteins, and branches in metabolism. In this model, there are 20 amino acids, each with a reversible enzyme that synthesizes (or imports) it, and a “ribosome” that synthesizes new proteins. This model does not include RNAs, so this ribosome does not need a template. The ribosome synthesizes new proteins in the desired proportions and with unequal proportions of the amino acids. We assume that the incorporation of each amino acid is a first-order kinetic process, so that the time is inversely proportionate to the concentration of the amino acid. The total time for a ribosome to translate a protein is then a weighted sum of the inverse concentrations, and so the rate of protein synthesis is proportionate to the concentration of ribosomes times the weighted harmonic mean of the amino acid concentrations. The rate of protein synthesis is equal to the growth rate.

**Fig. 4.**
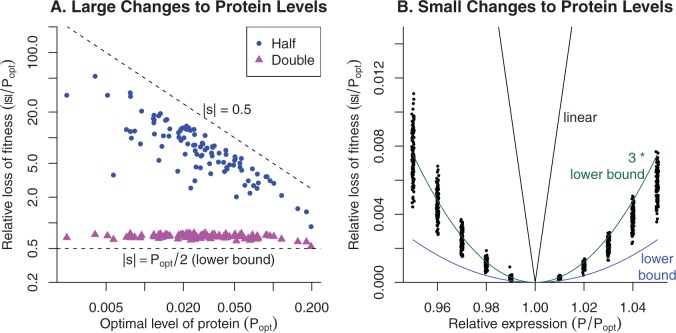
The impact of changes in expression levels in a simple growth model. In (A), we show the impact on fitness of changing a protein’s level by 2-fold away from its optimum (*P_opt_*) for each of 100 parameter settings. The impact on fitness is shown relative to *P_opt_*. Note log *x* and *y* axes. The two dashed lines shows our theoretical lower bound and the effect of a 2-fold reduction in the expression of a rate-limiting enzyme (|s|=0.5). In (B), we show the impact on fitness (*y* axis) of small relative changes in protein level (*x* axis). Again, the impact on fitness is relative to *P_opt_*, but the *y* axis is linear. We also show our theoretical lower bound ($s/P_{opt}=\epsilon^{2}$, where *ϵ* is the relative change in protein levels), 3 times that lower bound, and the linear relationship ($\left|s|/P_{opt}=|\epsilon \right|$).

We studied this model numerically, with a randomly selected range of parameters (see Models). We chose the parameters so that ribosomes had on average a 5-fold higher weight per unit activity than the enzymes. For each of 100 parameter settings, we identified the expression levels that maximized the growth rate. We will focus on the consequences of changing the expression of one of the enzymes away from this optimum, and we will assume that this enzyme is homomeric. (It does not matter whether the “ribosome” is comprised of a single protein or not, see Models.) As shown in figure 4a, in the growth model, doubling the enzyme’s expression reduced fitness by between $P_{opt}/2$ and *P_opt_*, which is similar to the cost-benefit model. In contrast, halving the enzyme’s expression had a strong effect on fitness (*s* = -0.02 to -0.32), which is far higher than in the cost-benefit model, and is closer to the rate-limiting step concept (which implies s=−0.5). Nevertheless, as shown in figure 4b, the fitness cost of small changes in relative expression, in either direction, was roughly quadratic in the fractional change. This quadratic was roughly three-fold higher than the lower bound that we obtained from metabolic models. But it was still far less than the linear relationship, |s|≈|ΔP|, which was proposed by Wagner (2005, 2007) as the reduction in fitness due to expression of excess protein. The fitness disadvantage of a small decrease in expression was also far less than implied by a rate-limiting step, which would give an even higher cost than the linear relationship (i.e., |s|≈|ΔP|/P).

As we mentioned previously, for linear pathways, the optimal expression level seems unrealistically high: $P_{opt}\approx \sqrt{K}$, where *K* is the expression level that gives the half-max benefit. The optimal protein expression was only moderately reduced in metabolic models with saturated enzymes or expensive intermediates (data not shown). In the growth model, we can define *K* in an analogous way to be the expression level that is below *P_opt_* and gives the half-max growth rate. We found that in the growth model, *P_opt_* is roughly 3·K (Supplementary fig. S1, Supplementary Material online). This is unrealistically low, as it implies that cutting *P* in half might have a strong impact on fitness. Indeed, in the growth model, the median reduction in fitness for cutting the enzyme level in half was 21%, which is far too high for most genes (Deutschbauer et al. 2005). A more realistic model that involved the assembly of multiple components and multi-step pathways for each component might yield an intermediate (and plausible) optimal level of expression.

## Disadvantage of Nonoptimal Expression in Metabolic Control Analysis

We then considered how to estimate the minimum cost of a change of expression for metabolic models more broadly. We used metabolic control analysis, which is based on a local linear approximation of the log flux or the log growth rate as a function of the log enzyme levels. This approximation has been used for a wide range of pathways including pathways with branches and cycles (Fell and Cornish-Bowden, 1997). The log-linear approximation might not be accurate, but intuitively it is hard to see why it would overstate the impact on fitness. Since we are interested finding a lower bound for the cost of changes to the expression, the log-linear approximation should be adequate.

We assume that the enzyme levels have evolved to maximize the flux, relative to a constraint on their total mass. Under the log-linear approximation, the flux is given by

$$F\propto \underset{i}{\Pi }P_{i}^{C_{i}}$$

where *F* is the flux, *P_i_* is the level of expression of protein *i*, and the parameter *C_i_* is referred to as the control coefficient. (The control coefficient is only approximately constant, and depends on the levels of metabolites and of other enzymes.) To maximize the flux, given a constrained total level of protein ($\sum P_{i}=1$), requires that $P_{i}\propto C_{i}$ (Brown, 1991).

For a purely metabolic system in which proteins act on metabolites but not on each other, and protein synthesis is ignored, the summation theorem (Kacser and Burns, 1981) states that

$$\sum_{i}C_{i}=1$$

To see why, consider that at steady state, you could double the level of every enzyme and keep the levels of all the metabolites the same. Since the net flux of a reaction is given by the enzyme concentration times a function of the metabolite concentrations, this will double all of the fluxes, but the system will still be at steady state and none of the metabolite concentrations will change. According to the formula, if every enzyme level doubles, *F* will increase by $2^{\left(\sum_{i}C_{i}\right)}$. So, this implies that the sum of the control coefficients *C_tot_* is 1.

Under these assumptions, the selective disadvantage of doubling a protein’s expression is roughly $0.31\cdot P_{opt}$, the selective disadvantage of halving expression is roughly $0.19\cdot P_{opt}$, and the selective disadvantage of a 1% change in expression is roughly 0.00005·P (see Models). These numbers are roughly 2-fold lower than in the cost-benefit form, which gave factors of 0.5, 0.5, and 0.0001, respectively. If the sum of the control coefficients is a bit higher than one, as might occur if proteins act on other proteins, then the selective effects increase proportionately. Overall, the log-linear approximation suggests that a wide range of metabolic models are consistent with disadvantages of around 0.2·P for 2-fold changes in expression and with tiny disadvantages for small changes in expression.

## Discussion

### Selection against Gene Duplications

The fitness disadvantage of changing a protein’s expression by 2-fold, or 0.5·P, may seem subtle, but this effect is likely to be significant. In both *E. coli* and *S. cerevisiae*, over 95% of proteins that have a measurable impact on fitness in a specific condition have $P>5\cdot 10^{-6}$ (Price et al. 2016; Deutschbauer et al. 2005; Ingolia et al. 2009). Thus, we have $s>0.5\cdot 5\cdot P=2.5\cdot 10^{-6}$, and if the effective population size is above 10^6^, then $N_{e}\cdot s>2$.

This also implies that many gene duplications will be selected against, as they will increase the total expression by 2-fold. One might imagine that gene regulation would adaptively correct the expression level of the gene, and reduce the impact of these changes on fitness. However, in *S. cerevisiae*, the expression level of most genes seems to respond directly to copy number, without any adaptive control (Springer et al. 2010). Because the majority of bacterial genes are not under direct adaptive control (Price et al. 2013), we expect this to be true in bacteria as well. For the genes that lack strong adaptive control, if the gene’s promoter region is duplicated along with the coding region, we expect that the duplication will usually be selected against.

Gene duplications are sometimes discussed as being beneficial because they improve tolerance to mutations, but the benefit is small, perhaps equal to the mutation rate (Walsh, 2003). For example, for a typical gene in *E. coli* of 1 kilobase, the total rate of mutations would be about 1,000 times the per-nucleotide mutation rate or $2\cdot 10^{-7}$ (Lee et al. 2012), which is much less than the disadvantage of $2\cdot 10^{-6}$ that we predict for duplicating a gene.

If selection on gene dosage is widespread, this will constrain the evolution of new gene functions. Genes’ functions often diverge after gene duplication, as one of the paralogs develops a new function or a new expression pattern, or the original function is subdivided between the two paralogs. Although the initial stage of this process is often described as being neutral, paralogs do not seem to evolve neutrally (Kondrashov et al. 2002). We propose that in microorganisms, gene duplications will only persist over evolutionary time if they support adaptation to a new environment (Kondrashov, 2012), for example by allowing the gene to be expressed in a new subset of conditions. This constraint could be part of why in bacteria, new protein functions usually evolve by horizontal gene transfer rather than by gene duplication within a lineage (Treangen and Rocha, 2011).

Our argument that gene duplications are selected against might seem paradoxical given that paralogs are widespread in microbial eukaryotes. However, in yeast, the rate of gene duplications is over 100-fold higher than had been estimated from evolutionary studies of retained paralogs (Lynch et al. 2008). This is consistent with the view that most duplications are selected against (Katju and Bergthorsson, 2013). We also note that, unlike prokaryotes, eukaryotes can undergo whole-genome duplication (WGD), as has occurred in *Saccharomyces* and in *Paramecium*. Because WGD does not alter the relative dosage of any gene, these duplications are neutral under our models. A final reason why paralogs might be more widespread in microbial eukaryotes than in bacteria, despite the selection against duplicated genes in both types of organisms, might relate to the cost of excess DNA. The reduction in fitness due to excess DNA is much smaller for larger cells (Lynch and Marinov, 2015), so that gene duplicates that are not expressed may persist in eukaryotes. In contrast, in bacteria, pseudogenes are removed by natural selection (Kuo and Ochman, 2010). If weakly-expressed paralogs are not selected against in eukaryotes, they would be more likely evolve a new and an adaptive expression pattern before they are lost by neutral decay.

### Selection on Small Changes to Expression

Our models suggest that, except for proteins in a tightly-bound heteromeric complexes, small changes in expression may not be under selection. Our metabolic models gave a lower bound of $|s|\approx \epsilon^{2}\cdot P$, where *ϵ* is the fractional change in the protein expression. In contrast, Wagner (2005, 2007) proposed that for an unnecessary increase in expression, |s|≈ϵ·P, which is dramatically higher. This high cost was derived by ignoring the incremental benefit of extra protein, and we propose that it is not appropriate for most genes. In our growth model, the fitness cost of a small change in expression was several times higher than in our metabolic models, but this is still far less than the high cost. For example, our models suggest that a small change in expression of 1% would have a selective disadvantage of between 0.0001·P and 0.0003·P. For a moderately expressed protein with $P=10^{-4}$, we estimate $s=10^{-8}$ to $3\cdot 10^{-8}$, which would be effectively neutral if $N_{e}<10^{7}$. However, the linear cost may be more appropriate for proteins that form stable heteromeric complexes. Also, it is easy to imagine that for regulatory proteins or signalling proteins, small changes in the expression could have larger effects than in our metabolic models. To better predict how sensitively fitness depends on expression levels, it would be interesting to build a kinetic model of a cell that included a realistic model of metabolism (Khodayari et al. 2014) as well as protein synthesis.

### Possible Relevance to Multi-Cellular Organisms

Although our models were developed with microorganisms in mind, our results may apply to some multi-cellular organisms. In larger organisms, selection can occur on fluxes such as the rate of carbon fixation (in plants) or the rate of energy production for movement (in animals), and the efficiency of these processes is analogous to our simple metabolic models. On the other hand, for many pathways, fitness might depend more on the efficiency of converting substrates into biomass, rather than the exact rate. It is not clear how perturbing enzyme levels would affect this sort of efficiency or whether the cost-benefit model would apply. However, the cost of a protein should be at least *P* if efficient production of useful biomass remains important. Given the metabolic control theory approach, this implies that a 2-fold change in expression will still have a fitness cost of at least 0.2·P.

If this is the case, then our models may explain why duplications of moderately-expressed genes are selected against in multi-cellular organisms that have relatively high effective population sizes. For example, the effective population size of *Drosophila melanogaster* is estimated to be around 10^6^ (Charlesworth, 2009), so the duplication of a gene that accounts for just $10^{-5}$ of the organism’s dry mass would be selected against ($N_{e}\cdot |s|>2$). Indeed, in *D. melanogaster*, gene duplications occur in the laboratory over 100 times faster than was expected from evolutionary comparisons (Katju and Bergthorsson, 2013).

## Conclusions

We predict that the selective disadvantage of changing a protein expression level by 2-fold should be at least 0.2·P, where *P* is that protein fraction of protein mass. This implies strong selection against the duplication of most microbial genes. Conversely, for most proteins, our models suggest that a small change in expression may be effectively neutral. Although the models of growth or metabolism that we considered are very simple, our prediction should be robust to complications such as saturating enzymes, branching pathways, multi-product reactions, or metabolic cycles. We did identify some exceptions. First, if the proteins are redundant, then selection on each individual protein expression will be weaker. Second, small changes in gene expression might not be neutral for proteins that form stable heteromeric complexes, or that produce or consume toxic metabolites, or for regulatory proteins.

## Models

### Derivation of the Cost-Benefit Form from a Linear Metabolic Pathway

The cost-benefit form can be justified by considering the optimization of a linear metabolic pathway that converts a substrate to a product (Waley, 1964; Heinrich and Schuster, 1998). Given parameters that describe the activities of the enzymes, and assuming that enzymes are not saturated by their substrates, the steady-state flux *F* can be written as

$$F\propto \frac{1}{\sum_{i}C_{i}/P_{i}}$$

where *P_i_* is the concentration of each enzyme and *C_i_* is related to the mass, per unit of activity, of each enzyme, or how costly that enzyme is (see equation 8 of (Heinrich and Schuster, 1998)).

We focus on the expression of one enzyme at level *P* and assume that the expression of all the other enzymes varies in proportion to 1−P. If we choose appropriate parameters, then we obtain a steady-state growth rate of

$$F\propto \frac{1}{C_{1}/P+C_{2}/(1-P)}\propto \frac{P}{K+P/(1-P)}$$

where $K=C_{1}/C_{2}$. (For simplicity, we show the derivation for a 2-step pathway, but it holds for longer linear pathways as well.) This “metabolic” form is approximately the same as the cost-benefit form with *f* = 1 when K≪1, as is the case for virtually all genes. (At the optimal expression, an essential gene with K = 0.01 would be 9% of cellular protein.)

The optimal values under this “metabolic” form are

$$P_{opt}=\frac{\sqrt{K}}{1+\sqrt{K}}$$

$$s_{opt}=\frac{1}{{\left(1+\sqrt{K}\right)}^{2}}$$

while the cost-benefit form gives

$$P_{opt}=\sqrt{K\cdot f}-K$$

$$s_{opt}={\left(\sqrt{f}-\sqrt{K}\right)}^{2}$$

Setting *f* = 1 and using a Taylor expansion in terms of $\sqrt{K}$ around zero shows that the two forms give very similar results. At P≈0.1 and *K* = 0.01, *s_opt_* and *P_opt_* have fractional differences of just 1-2%. We also verified that the impact of changing a protein’s expression away from the optimum is similar to the metabolic form as for the cost-benefit form with *f* = 1.

In the cost-benefit form, if the expression level changes away from its optimum by a small fraction ϵ, so that $P=P_{opt}\cdot (1+\epsilon )$, then the change in fitness is roughly

$$s_{\epsilon }\approx -\epsilon^{2}\cdot P_{opt}/(1+\epsilon )$$

### Simulations of Reversible Michaelis-Menten Kinetics

For each parameter setting in figure 3, we considered $P=5\cdot 10^{-4}$ to 0.5, stepping by $5\cdot 10^{-4}$. For each value of *P*, we solved numerically for the steady state concentration of the intermediate *I* (so that the flux from *S* to *I* equals the flux from *I* to *E*). We used reversible Michaelis-Menten kinetics, in which the flux from *S* to *I* is

$$\frac{\left(S-I/K_{eq1})\cdot (V_{1}/K_{1f}\right)}{\left(1+S/K_{1f}+I/K_{1r}\right)}$$

where $K_{eq1}$ is the equilibrium constant for S⟷I, $K_{1f}$ and $K_{1r}$ are saturation constants, and *V*_1_ is the enzyme activity. We used $V_{1}=100\cdot P$ and, for the second step, $V_{2}=1-P$.

### Simulations of a Growth Model

For simulations with 20 amino acids being assembled into proteins by a “ribosome”, we assumed that the growth rate is the same as the rate at which the ribosome makes new proteins:

$$g=\frac{R}{\sum_{i}f_{i}/A_{i}}$$

where *R* is the concentration of ribosomes, *f_i_* is the fraction of amino acid *i* in proteins, and *A_i_* is the concentration of amino acid *i*. Notice that the rate of the ribosome is implicitly set to 1 and that each amino acid is incorporated at the same rate relative to its concentration. The rates for the other enzymes are assumed to be scaled relative to the rate of the ribosome. We assumed unsaturated reversible kinetics of the enzymes for synthesizing amino acids, with a substrate concentration of 1, an equilibrium constant of 10, a rate constant *r_i_*, and a concentration *E_i_*.

To simplify the fitting of this model, we focused on the enzyme for making one amino acid, and we gave the other 19 amino acids equal values for the parameters *f_i_* and *r_i_*. We considered 100 random settings of the parameters *f_i_* and *i_i_*. The amino acid usage of the focal amino acid (*f*_1_) was distributed as $2^{N_{1}}/\sum 2^{N_{i}}$, where *N_i_* are 20 standard normal variables. *f*_1_ ranged from 0.011 to 0.407 (median 0.043). For $i>1,f_{i}=(1-f_{1})/19$. The enzyme rates followed the same distribution, but were scaled so that the average value was 5, so that the enzymes were typically faster (or lighter) than the ribosome.

At steady-state, the production and consumption of each amino acid is constant, so $g\cdot f_{i}=E_{i}\cdot r_{i}\cdot (1-A_{i}/10)$. Given the protein concentrations, we solved numerically for the steady-state amino acid concentrations and hence the growth rate. Specifically, we used the nlm function in R to minimize the square root of the total squared deviation from equal consumption and production. We used multiple starting points to ensure convergence to very low deviation. We used a higher-level numerical optimization (again with nlm) to maximize the growth rate, subject to the constraint that the total protein concentration $R+\sum_{i}E_{i}=1$. Because of the symmetry in the parameters for amino acids 2 through 20, we assumed that $E_{2}=E_{3}=\ldots =E_{20}$. The optimal expression level of the focal enzyme ranged from 0.002 to 0.197 (median 0.026). Given the optimal protein levels, we then calculated the selective disadvantage (the reduction in the relative growth rate) of changes in protein levels.

Given that we focused on deviations in the expression of one (homomeric) enzyme, it makes no difference if the other enzymes or the ribosome have multiple subunits. This is because the expression of all other proteins is assumed to change proportionately as the focal enzyme’s expression changes. Because all subunits’ expression would change in unison, the concentration of active enzyme or ribosome would change in the expected way. Although this assumption is plausible, it might not be accurate if some subunits’ transcripts have a stronger affinity for the ribosome than do other transcripts.

### The Disadvantage of Nonoptimal Expression in a Metabolic Control Analysis Model

Given the log-linear approximation, the flux *F* is given by

$$F\propto \underset{i}{\Pi }P_{i}^{C_{i}}$$

where *P_i_* is the concentration of each protein and *C_i_* are the control coefficients. We also assume the summation theorem:

$$\sum_{i}C_{i}=1$$

If some of the proteins are replaced by useless proteins, so that every protein is reduced in concentration by a small fraction *f_U_*, then the new growth rate is given by

$$g(f_{U})=g(f_{U}=0)\cdot {\left(1-f_{U}\right)}^{C_{tot}}=g(f_{U}=0)\cdot (1-f_{U})$$

so that the cost of expressing a useless protein equals the fraction of protein that it accounts for. If the total control coefficient is greater than one, as might occur if proteins act on other proteins, the cost will be a power of $1-f_{U}$ but the trend will be similar. Also note that if the cost of useless protein is at least equal to its expression level, then the total control coefficient must be at least one.

Now, let us vary the expression of one protein, and assume that the expression of the other proteins varies proportionately to keep the total level of protein constant. In that case, the above formulation of the growth rate simplifies to $F\propto P^{C}\cdot {\left(1-P\right)}^{C_{tot}-C}$. If the total control coefficient is 1, then the optimal expression level is *P*=* C*. For P≪1, the effect on fitness of a change in expression from *P* to (1+ϵ)·P is very close to

$$s_{\epsilon }\approx (-\epsilon +\log(1+\epsilon ))\cdot P_{opt}$$

which yields a selective disadvantage of $0.31\cdot P_{opt}$ for doubling expression, $0.19\cdot P_{opt}$ for halving expression, and roughly $0.5\cdot \epsilon^{2}$ for small changes in expression. If *C_tot_* > 1, then the selective disadvantage of higher-than-optimal expression is increased in proportion to *C_tot_*, so modest deviations from the assumption that $\sum_{i}C_{i}=1$ will not make much difference.

## Supplementary Material

Supplementary Data

## Supplementary Material

**Supplementary** figure S1 is available at *Genome Biology and Evolution* online (http://www.gbe.oxfordjournals.org/).
