# Supplementary material for: A Theoretical Lower Bound for Selection on the Expression Levels of Proteins
Source: Genome Biol Evol. 2016 Jun 11;8(6):1917–28. doi: 10.1093/gbe/evw126 (PMC4943197; doi:10.1093/gbe/evw126)
Supplement: Supplementary Data [file supp_8_6_1917__index.html]

A Theoretical Lower Bound for Selection on the Expression Levels of Proteins — Supplementary Data 

# A Theoretical Lower Bound for Selection on the Expression Levels of Proteins

## Supplementary Data

files

- Supplementary Data - pdf file
