# Supplementary material for: A Theoretical Lower Bound for Selection on the Expression Levels of Proteins
Source: Genome Biol Evol. 2016 Jun 11;8(6):1917–28. doi: 10.1093/gbe/evw126 (PMC4943197; doi:10.1093/gbe/evw126)
Supplement: Supplementary Data [file supp_evw126_dup_S1.pdf]

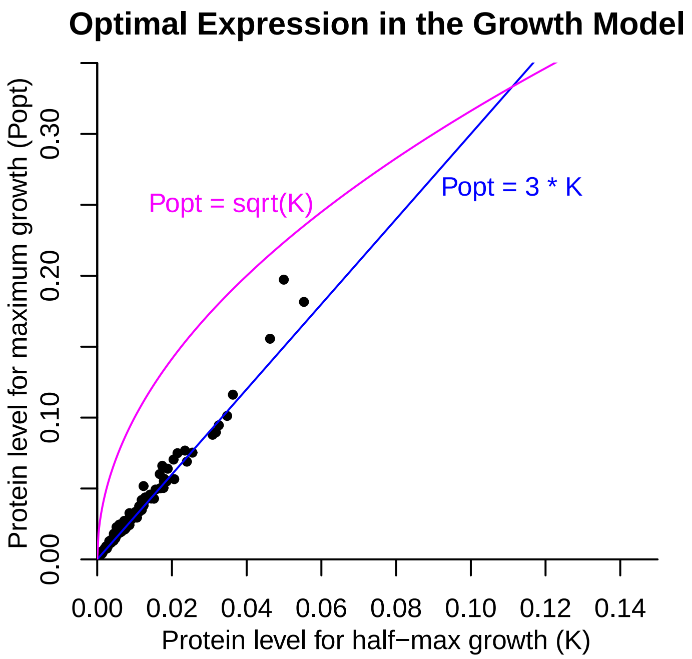


**Supplementary Figure S1: Optimal expression ($P_{opt}$) versus expression for half-max growth ($K$) in the growth model.**
